# Zoospore interspecific signaling promotes plant infection by *Phytophthora*

**DOI:** 10.1186/1471-2180-10-313

**Published:** 2010-12-07

**Authors:** Ping Kong, Brett M Tyler, Patricia A Richardson, Bobby WK Lee, Zhaohui S Zhou, Chuanxue Hong

**Affiliations:** 1 Department of Plant Pathology, Physiology and Weed Science, Virginia Polytechnic Institute and State University, Virginia Beach, VA 23455-3363, USA; 2 Virginia Bioinformatics Institute, Virginia Polytechnic Institute and State University, Blacksburg, VA 24061-0477, USA; 3 The Barnett Institute and Department of Chemistry and Chemical Biology, Northeastern University, Boston, MA 02115, USA

## Abstract

**Background:**

Oomycetes attack a huge variety of economically and ecologically important plants. These pathogens release, detect and respond to signal molecules to coordinate their communal behaviors including the infection process. When signal molecules are present at or above threshold level, single zoospores can infect plants. However, at the beginning of a growing season population densities of individual species are likely below those required to reach a quorum and produce threshold levels of signal molecules to trigger infection. It is unclear whether these molecules are shared among related species and what their chemistries are.

**Results:**

Zoospore-free fluids (ZFF) from *Phytophthora capsici*, *P. hydropathica*, *P. nicotianae *(ZFFnic), *P. sojae *(ZFFsoj) and *Pythium aphanidermatum *were cross tested for stimulating plant infection in three pathosystems. All ZFFs tested significantly increased infection of *Catharanthus roseus *by *P. nicotianae*. Similar cross activities were observed in infection of *Lupinus polyphyllus *and *Glycine max *by *P. sojae*. Only ZFFnic and ZFFsoj cross induced zoospore aggregation at a density of 2 × 10^3 ^ml^-1^. Pure autoinducer-2 (AI-2), a component in ZFF, caused zoospore lysis of *P. nicotianae *before encystment and did not stimulate plant infection at concentrations from 0.01 to 1000 μM. *P. capsici *transformants with a transiently silenced AI-2 synthase gene, ribose phosphate isomerase (*RPI*), infected *Capsicum annuum *seedlings at the same inoculum concentration as the wild type. Acyl-homoserine lactones (AHLs) were not detected in any ZFFs. After freeze-thaw treatments, ZFF remained active in promoting plant infection but not zoospore aggregation. Heat treatment by boiling for 5 min also did not affect the infection-stimulating property of ZFFnic.

**Conclusion:**

Oomycetes produce and use different molecules to regulate zoospore aggregation and plant infection. We found that some of these signal molecules could act in an inter-specific manner, though signals for zoospore aggregation were somewhat restricted. This self-interested cooperation among related species gives individual pathogens of the same group a competitive advantage over pathogens and microbes from other groups for limited resources. These findings help to understand why these pathogens often are individually undetectable until severe disease epidemics have developed. The signal molecules for both zoospore aggregation and plant infection are distinct from AI-2 and AHL.

## Background

Zoosporic plant pathogens in the phylum Oomycota of the Stramenopila kingdom include hundreds of devastating species that attack a broad range of economically important agricultural and ornamental crops as well as forest tree species [1,2]. These oomycetes, including *Phytophthora *and *Pythium *species, use motile zoospores for dispersal and plant infection [3-5]. Plant infection by zoosporic pathogens is often effective in nature despite the fact that the population density in primary inoculum sources is relatively low [6-9]. This has led to differing theories with regard to density-dependent zoospore behaviors and plant infection [10-17]. A recent study with *Phytophthora nicotianae *showed that plant infection may be regulated through zoosporic extracellular products in zoospore-free fluid (ZFF) which can promote infection by a single zoospore [18]. This indicates that the physical presence of the threshold density of zoospores at an infection site is not strictly required, and plant infection can be initiated efficiently through chemical communication by the population. However, it is not clear how such a process is carried out by a pathogen at its naturally occurring low population density, which would be unlikely to produce adequate levels of functional signals unless these signals were also produced by other organisms and readily accessible in the environment.

Ca^2+ ^and autoinducer 2 (AI-2), two widespread and non-specific signaling molecules, are known to be produced by zoosporic oomycetes [19-21]. Ca^2+ ^plays a central role in autonomous encystment, adhesion and germination of cysts in zoosporic oomycetes [3,10,14,22-24]. However, it is not considered to be an autoinducer because Ca^2+ ^does not directly trigger cooperative behaviors of zoospores and acts more like a secondary messenger [18]. AI-2 was first detected in bacteria and is utilized for metabolism and quorum sensing in bacteria [25-27]. In the latter process, bacteria respond to these released signaling molecules or autoinducers to coordinate their communal behavior. Eukaryotes including oomycetes can also produce AI-2 or AI-2-like activities [21,28-30] although they do not use the LuxS pathway that most bacteria use [31,32]. Instead, AI-2 is formed spontaneously from D-ribulose-5-phosphate that is synthesized in these eukaryotes from pentose-phosphates by ribose phosphate isomerase (RPI) in the pentose-phosphate pathway [28]. AI-2 has been proposed as a universal signaling molecule in bacteria based on its role in inter-species signaling and postulated cross-kingdom communication [33-40]. However, the function of AI-2 in eukaryotes has not been established.

The aim of this study was to investigate the nature of signal molecules in ZFF. Specifically, we identified inter-specific signaling activities of ZFF from four *Phytophthora *species and one *Pythium *species. We also assessed the potential of AI-2 along with another known bacterial autoinducer as signal molecules for communication among zoosporic species.

## Results and Discussion

### ZFF interspecific stimulation of zoosporic infection

**Figure 1:**
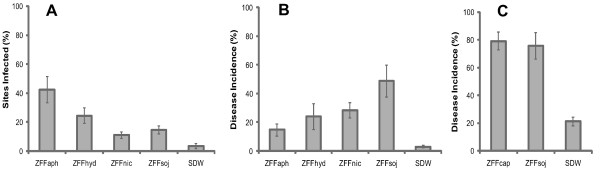
**Cross effects of zoospore-free fluid **(**ZFF) from different pythiaceous species on plant infection by *Phytophthora *sp**. ZFF was prepared from zoospore suspensions of *Py. aphanidermatum *(ZFFaph) and *P. hydropathica *(ZFFhyd) at 3 × 10^4 ^ml^-1^, and from *P. capsici *(ZFFcap), *P. nicotianae *(ZFFnic) and *P. sojae *(ZFFsoj) at 5 × 10^4 ^ml^-1^, respectively. Each ZFF was used as diluent to prepare inocula at a final density of 100 zoospores ml^-1 ^(or approximately 1 per 10-μl drop) and evaluated against sterile distilled water (SDW) in three pathosystems. (A) *Catharanthus roseus *cv. Little Bright Eye × *P. nicotianae*. Ten drops of inoculum were applied to the underside of each detached leaf at different sites and infection was assessed after 3-day incubation at 23°C. Each column is a mean percentage of sites diseased (N = 54). (B) *Lupinus polyphyllus *× *P. sojae*. Two drops of inoculum were applied to each cotyledon and disease was assessed after 5-day incubation at 23°C. Each column is a mean percentage of dead seedlings (N = 30). (C) *Glycine max *cv. Williams × *P. sojae*. Two drops of inoculum were applied to hypocotyl of each seedling and disease was assessed after 4-day incubation at 26°C. Each column is a mean percentage of dead seedlings (N = 6). Bars represent standard deviation in each case.

Zoospore-free fluids were prepared from suspensions at a density of 10^4 ^zoospores ml^-1 ^or higher of *Phytophthora nicotianae *(ZFFnic), *P. capsici *(ZFFcap), *P. hydropathica *(ZFFhyd), *P. sojae *(ZFFsoj) and *Pythium aphanidermatum *(ZFFaph) and evaluated in three phytopathosystems. Inoculation of annual vinca (*Catharanthus roseus*) with suspensions containing an average of one zoospore of *P. nicotianae *in any of the four ZFFs resulted in significantly higher infection (*P *< 0.001) compared to the control (SDW). Specifically, percentages of sites infected were 39%, 21%, 11%, and 15% for ZFFaph_, _ZFFhyd, ZFFnic, and ZFFsoj, respectively compared to 3% for SDW (Figure 1A). Similarly, ZFFaph, ZFFhyd, ZFFnic and ZFFsoj stimulated infection of lupine (*Lupinus polyphyllus*) by *P. sojae *(Figure 1B), while ZFFcap and ZFFsoj stimulated infection of soybean (*Glycine max*) by *P. sojae *(Figure 1C). These results indicate that ZFF from the different *Phytophthora *species and *Py. aphanidermatum *contained one or more signals stimulating zoosporic infection by *P. nicotianae *and *P. sojae *that are active across species boundaries.

Many plants are attacked by multiple oomycete species [1]. The ability of oomycete pathogens to benefit from the presence of related (or unrelated) species is presumably a selective advantage, especially if the diverse pathogens are competing for a limited resource (i.e. the host plant tissue) and/or the initial population density of each individual pathogen population is low. Such self-interested cooperation may have further advantages if the effector molecules released by each pathogen species have complementary or synergistic capabilities for suppressing plant defenses.

### ZFF inter-specific regulation of zoospore aggregation

**Figure 2:**
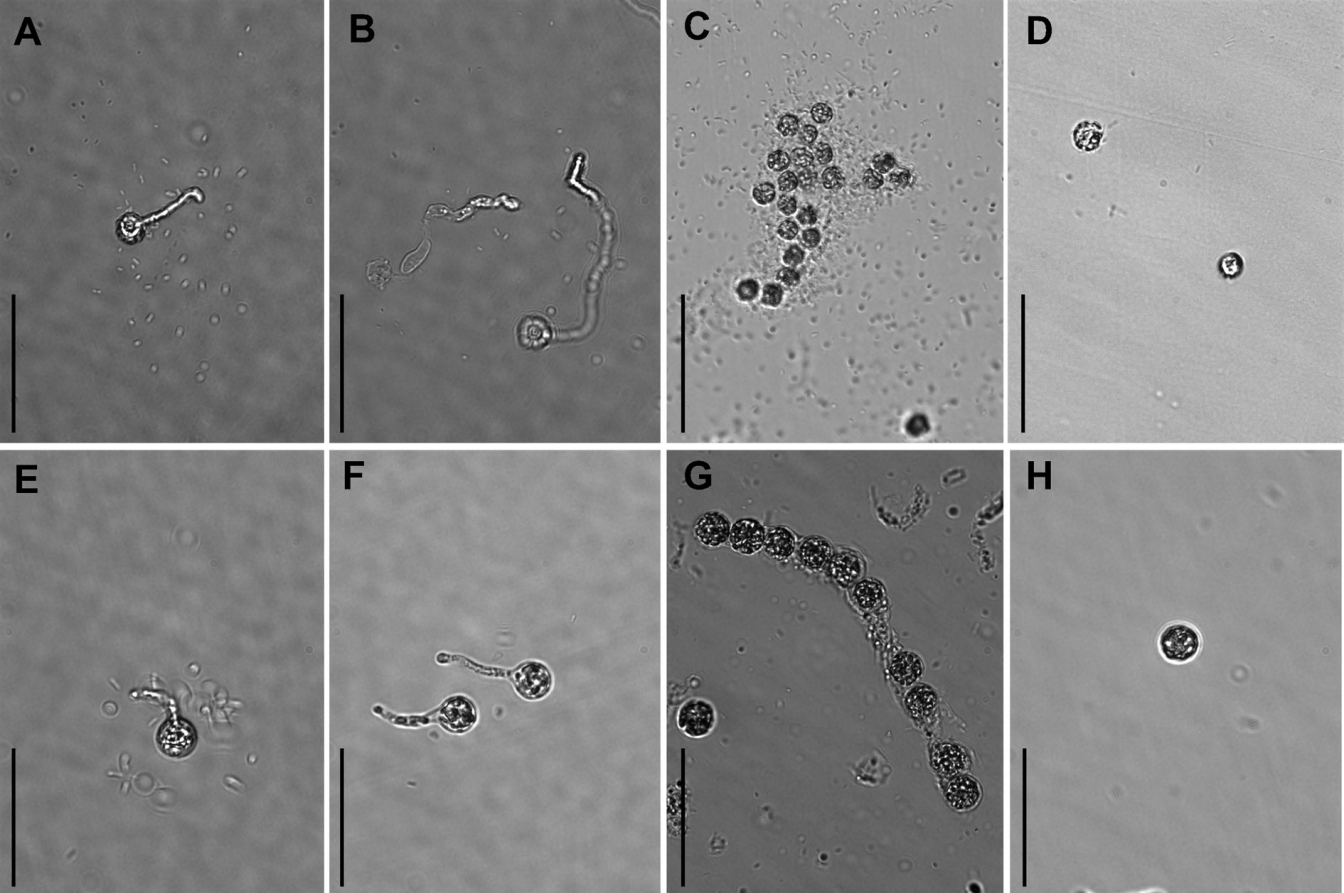
**Effect of zoospore-free fluid (ZFF) on aggregation of *Phytophthora nicotianae *and *Phytophthora sojae *zoospores**. Zoospores of *P. nicotianae *(2 × 10^3 ^ml^-1^) were incubated in ZFF of (A) *Py. aphanidermatum*, (B) *P. capsici*, (C) *P. sojae*, and (D) sterile distilled water (SDW). Zoospores of *P. sojae *(2 × 10^3 ^ml^-1^) were incubated in ZFF of (E) *Py. aphanidermatum*, (F) *P. capsici*, (G) *P. nicotianae *and (H) SDW. Images were captured 18 hours after incubation at 23°C. Bar = 50 μm.

To determine whether ZFF may also have cross-species activity in regulating zoospore aggregation, fresh zoospores of *P. nicotianae *and *P. sojae *at a concentration (2 × 10^3 ^ml^-1^) below normal aggregation thresholds (approx. 10^6 ^ml^-1^) were cross incubated in multiwell plates with ZFFsoj or ZFFnic and compared with those in SDW. Zoospores of *P. nicotianae *in ZFFsoj and those of *P. sojae *in ZFFnic aggregated (Figure 2C and 2G) as if they were in ZFF produced by their own species. As expected, zoospores of neither species aggregated in SDW (Figure 2D and 2H). ZFFcap and ZFFaph did not stimulate zoospore aggregation by *P. nicotianae *or *P. sojae *zoospores. However, they did stimulate germination of cysts of both *P. nicotianae *and *P. sojae *(Figure 2A, B, E, F), which may explain their activity in promoting plant infection (Figure 1). It was interesting that zoospores of *P. capsici *did not aggregate even at a density of 10^6 ^zoospores ml^-1^. These results indicate that the signal(s) involved in aggregation are somewhat species-restricted and may be different from those mediating the infection process.

### AI-2 is not involved in zoospore communication and promotion of plant infection

To test whether AI-2 may be involved in zoospore communication and promotion of plant infection, purified AI-2 was used in place of ZFF. AI-2 was tested at a wide concentration range of 0.01 μM -1 mM for its effects on *P. nicotianae *zoospore behaviors and plant infection; the concentration of AI-2 in ZFF was estimated to be less than 2 μM [21]. Under the microscope, an increased number of zoospores treated with AI-2 lysed before encystment and failed to germinate as the AI-2 concentration was increased (Table 1). Zoospore aggregation was not observed at any concentration tested. In infection experiments with annual vinca, AI-2 did not promote single zoospore infection at any concentration. Interestingly, AI-2 induced hypersensitive response (HR)-like micro-lesions on the inoculated sites at 100 μM and higher. These results indicated that AI-2 was not responsible for any of the zoospore signals found in ZFF.

**Table 1:** Effect of purified AI-2 on encystment and germination of P. nicotianae zoospores after overnight incubation at 23°C

Conc. of AI-2 (μM)	No. of cysts		No. of germinating cysts		No. of empty cells		No. of lysed **zoospores**^**a**^	
	**M**^**b**^	**Std**^**b**^	**M**	**Std**	**M**	**Std**	**M**	**Std**
	
0	5	0.3	12	2.3	39	1.0	1	3.8
0.01	10	0.3	7	0.5	22	1.3	17	1.0
0.1	5	0.5	4	0.8	22	0.8	25	0.5
1	2	0.3	0	0.0	21	1.8	33	2.0
10	11	0.5	0	0.0	22	2.1	19	2.5
100	20	1.0	0	0.0	0	0.0	36	1.0
1000	14	1.3	0	0.0	0	0.0	42	1.3

^a ^Difference between the total number of zoospores (56 ± 4) in SDW and those countable in AI-2 at each concentration.

^b ^M is the mean from 12 replicate fields (at 100×) of three assays. Std is the standard deviation.

**Figure 3:**
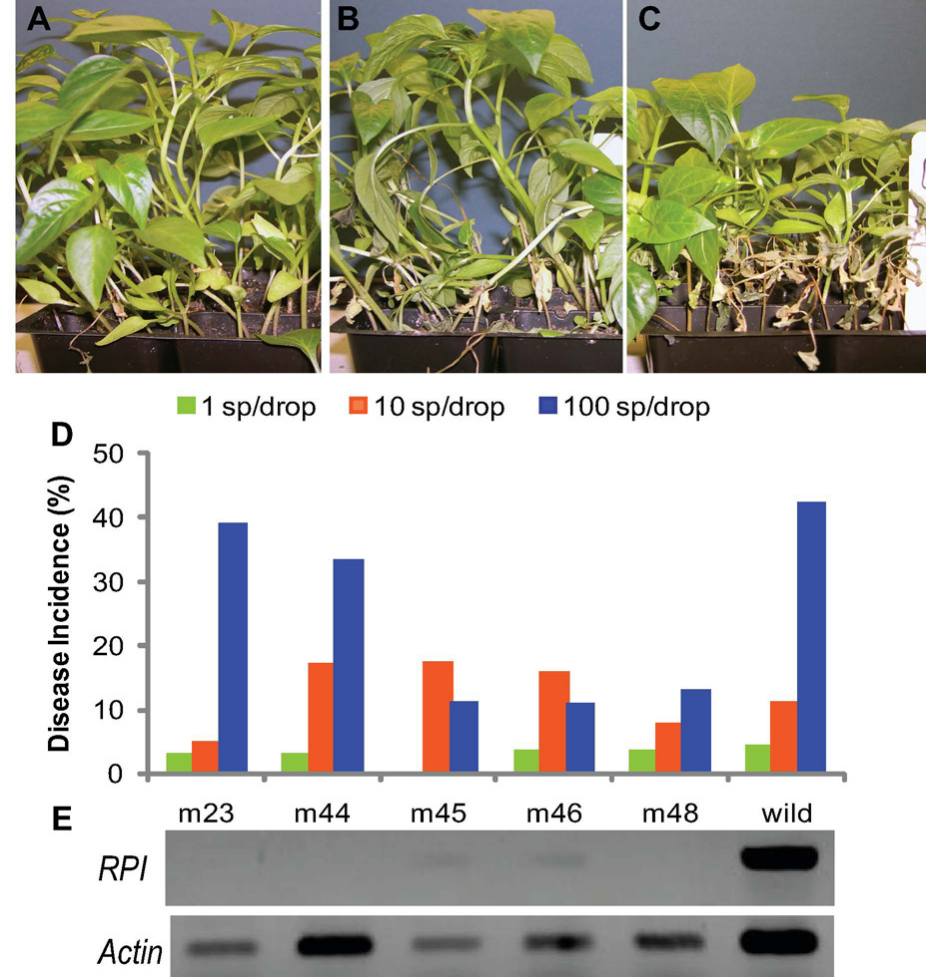
**Infection of *Capsicum annuum *cv. California Wonder by wild or gene-silenced *Phytophthora capsici***. Two 10-μl drops of zoospore suspension at 10^2^, 10^3 ^or 10^4 ^ml^-1 ^were applied to hypocotyl of pepper seedling and disease was assessed after 5-day incubation at 26°C. (A, B, C) Symptoms on seedlings inoculated with wild type at 10^2^, 10^3 ^and 10^4 ^zoospores ml^-1^, respectively. (D) Disease incidence of seedlings inoculated with wild or ribose phosphate isomerase (RPI) gene-silenced strains (N = 6). (E) *RPI *expression in transiently silenced lines (T_1_) on day 14 after transfer from7 day- old regenerated transformants (T_0_) treated with dsRNA as indicated by the RT-PCR products of *RPI *compared with equal amounts of endogenous control *actin *from the T_1 _mutant RNA.

As a complementary test for the ability of AI-2-like molecules to mediate zoospore communication and promote plant infection, we cloned and silenced the ribose phosphate isomerase (RPI) gene of *P. capsici*. RPI converts ribose-5-phosphate to ribulose-5-phosphate, which can spontaneously convert to AI-2-like molecules under physiological conditions [28]. RPI was proposed to be responsible for production of AI-2-like molecules in zoosporic pathogens [21]. To silence the *RPI *gene of *P. capsici*, protoplasts of *P. capsici *were treated with *RPI *dsRNA. If RPI had a role in production of zoospore signaling molecules, *RPI*-silenced lines would be expected to require much higher zoospore concentrations to infect plants than the wild type due to reduced or blocked AI-2 production by the inocula. One third of the 48 T_0 _lines regenerated 7 days after dsRNA exposure showed no or decreased expression with *RPI *compared to the endogenous control *actin *detected using RT-PCR. Half of these silenced or down regulated *RPI *lines retained the same reduced transcript levels two weeks after being transferred to fresh media (T_1_) (Figure 3E). Five T_1 _lines were simultaneously tested for zoospore threshold for infection. The resulting disease incidences were very similar to those produced by wild type *P. capsici *at zoospore inoculum concentrations ranging from 10^2 ^to 10^4 ^ml^-1 ^(Figure 3A-D) (*P *= 0.705; *P *= 0.065; *P *= 0.598, respectively). These results indicate that *RPI *silencing had no significant impact on zoospore communication during infection. The ZFF activity of the silenced lines was not evaluated due to the transient nature of dsRNA-mediated silencing [41] and insufficient numbers of T_1 _zoospores for ZFF production. Nevertheless, these findings are consistent with the conclusion that AI-2-like molecules that might be produced via the action of RPI are not required for infection at low inoculum densities.

The function of AI-2-like activities produced by zoosporic oomycetes remains unclear although it regulates bacteria quorum sensing [21]. Two-way communication has been observed between eukaryotes and bacteria such as *Leguminosae *and bacterial rhizobia [42] and between mycorrhiza and *Streptomyces *[43]. In the former case, plants release flavonoids that bind LysR-family transcriptional regulators in the bacteria, leading to the production of Nod factor that facilitates nitrogen fixation. In the latter case, fungal metabolites stimulate the bacteria to produce auxofuran which promotes growth of both the fungus and the host plants. Perhaps zoosporic oomycetes utilize AI-2 to attract quorum sensing bacteria which subsequently release factors that facilitate plant infection. Indeed, bacteria have been shown to benefit sporangium production by zoosporic oomycetes [44].

### Involvement of other molecules in ZFF activity

Acyl-homoserine lactones (AHLs), or bacterial autoinducer 1, are utilized by zoospores of the green seaweed *Enteromorpha *(*Ulva*) for communication in the search for settlement surfaces [45]. A bioassay was performed using the *Agrobacterium tumefaciens *reporter strain KYC55/pJZ410/pJZ384/pJZ372 [46] in plate and spectrophotometric tests to determine whether this molecule is present in ZFF. LacZ activity was detected in all four positive control plates at nM concentrations of AHL but not in ZFFnic or ZFFsoj prepared from zoospore suspensions at > 10^4 ^spores ml^-1 ^nor in concentrated extracts from them obtained with ethyl acetate. These results indicate that zoospores from these oomycete species do not produce AHLs which therefore cannot be responsible for any ZFF activity.

### Temperature sensitivity of ZFF activities

**Figure 4:**
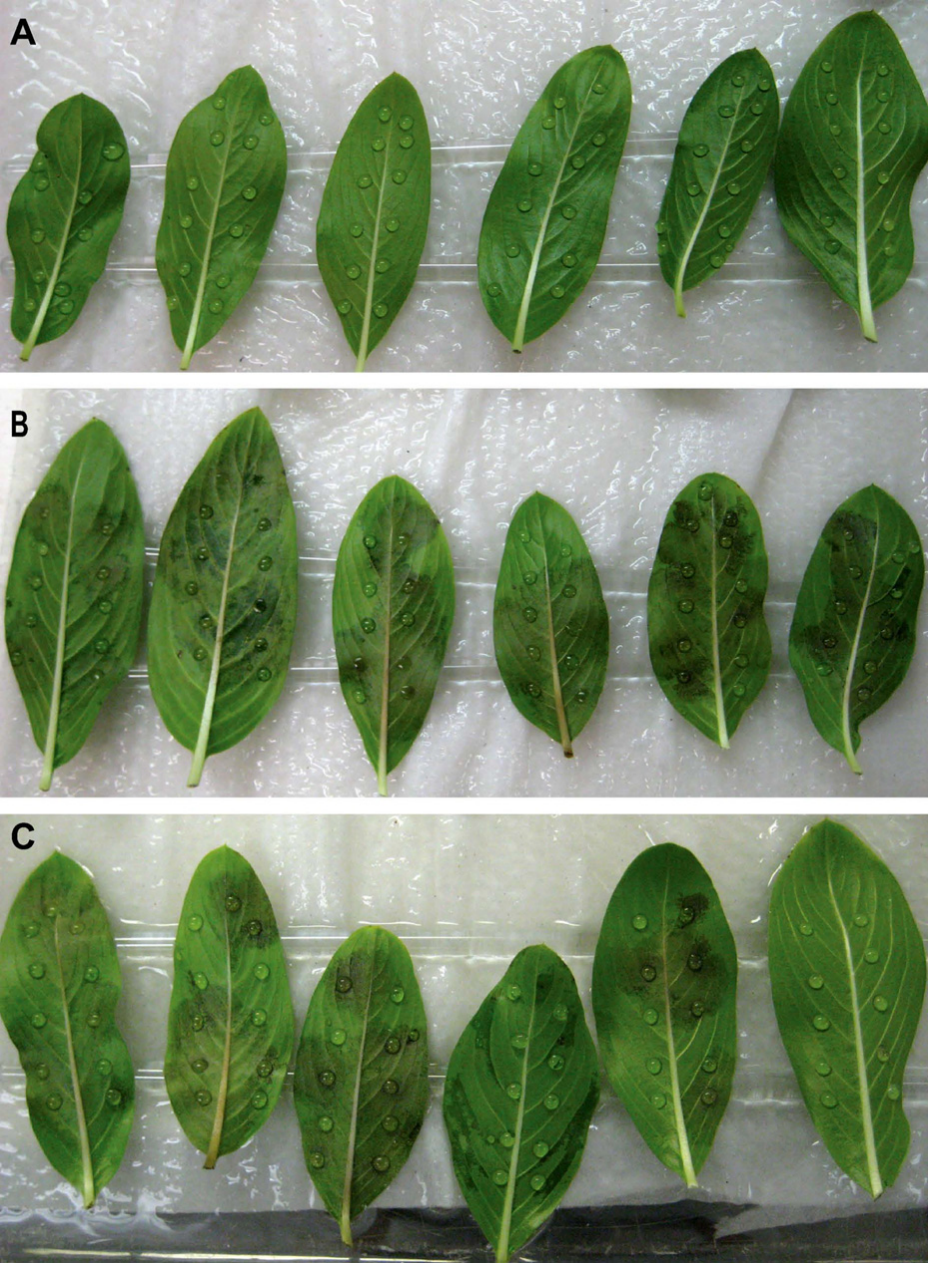
**Zoospore-free fluid (ZFF) stimulation of *Phytophthora *infection is unaffected by heat treatment**. Each leaf of *Catharanthus roseus *cv. Little Bright Eye was inoculated with twelve 10-μl drops of inoculum of *P. nicotianae *at approximately one zoospore per drop. Zoospores were suspended in (A) sterile distilled water, (B) untreated ZFF from the same species at 5 × 10^5 ^zoospores ml^-1 ^and (C) heat-treated ZFF. Disease symptoms were photographed after 3-day incubation at 23°C.

To begin to characterize the signal molecules in ZFF we tested their temperature sensitivity. ZFFnic did not stimulate zoospore aggregation after a freeze-thaw or heat treatment, suggesting that the molecule promoting this behavior may be a protein or lipoprotein that is sensitive to heat and freezing. On the other hand, freeze-thaw did not affect the activity of ZFFnic in promoting plant infection by zoospores (data not shown). In addition, ZFFnic boiled for 5 minutes remained as active as the untreated in promoting infection (Figure 4), indicating that the molecule which stimulates plant infection is temperature insensitive and different from that involved in aggregation.

## Conclusion

This study demonstrated inter-specific activities of zoospore extracellular products in promoting zoospore aggregation and plant infection by *Phytophthora*. Zoosporic oomycetes contain hundreds of species and are widespread in irrigation water and plant production fields. However, specific populations detected in primary inoculum sources are not in sufficient numbers to produce signals that could promote plant infection. Inter-specific chemical communication (probably self-interested) as a strategy used by zoosporic pathogens for effective plant infection provides insights into the destructiveness of these pathogens and the importance of the microbial community and the environment in the infection process.

AI-2 was excluded as a signal for communal behavior in zoosporic oomycetes, despite its detection in ZFF and widespread presence in the environment. AI-2 synthase RPI and purified AI-2 both were not required for regulation of zoospore aggregation and infection. AHLs also were excluded because of their absence in ZFF. Thus, zoosporic oomycetes may use completely different chemicals from bacteria for quorum sensing. Analysis of ZFF revealed that functional signals controlling zoospore aggregation and plant infection differ in molecular composition. The former is not temperature labile and acts upon a restricted number of species while the latter is heat labile and non-species-specific. Identifying these molecules will facilitate our understanding of the mechanisms underlying natural plant infection by these pathogens and may lead to innovative control strategies.

## Methods

### Zoosporic oomycetes and culture conditions

Four *Phytophthora *species, *P. nicotianae *(1B11), *P. sojae *(28G4), *P. capsici *(24F4), *P. hydropathica *(37E6) and one *Pythium *species *Py. aphanidermatum *(18H7) were used in this study. These species are distinct in morphology and genetics [2,47]. Specifically, *P. nicotianae*, *P. capsici *and *Py. aphanidermatum *have broad host ranges while *P. sojae *has a restricted host range, generally infecting only soybeans and lupines. *P. hydropathica *(37E6) originated from irrigation water and is a pathogen of nursery plants [48]. The isolates were maintained on clarified vegetable juice agar (CV8A) medium [49] at 23°C.

### Preparation of zoospore-free fluid

Zoospore-free fluid (ZFF) from a particular species is designated with an abbreviated species name. For example, ZFFnic represents ZFF from a *P. nicotianae *zoospore suspension. ZFF was prepared from nutrient-depleted zoospore suspensions starting with sporangium induction as described previously [18,21]. Specifically, prior to sporangium production, *P. sojae *and *Py. aphanidermatum *were cultured for 3-4 days and the other species were cultured for 1-2 wk in 10% CV8 broth. After nutrient depletion (medium removal and water rinses), the mycelial mats were further incubated for 16-18 h for *P. sojae *and *Py. aphanidermatum*, 2-3 days for *P. capsici *and one week for the other species under fluorescent light at 23°C to obtain a desired number of sporangia. To induce zoospore release, the mats with sporangia were flooded with chilled SDW and kept under lights until the desired zoospore density was reached. ZFF was obtained by passing a zoospore suspension through a 0.2 μm pore-size filter after vortexing for 2 min. ZFF was used fresh or stored at -20°C. Freezing destroyed the aggregation-promoting activity of ZFF, but not its infection-promoting activity.

### Phytopathosystems, plant growth conditions, inoculum preparation and inoculation

Four phytopathosystems, *P. nicotianae *× annual vinca (*Catharanthus roseus *cv. Little Bright Eye), *P. sojae *× lupine (*Lupinus polyphyllus*), *P. sojae *× soybean (*Glycine max *cv. Williams) and *P. capsici *× pepper (*Capsicum annum *cv. California Wonder) were used.

Annual vinca plants were prepared in the greenhouse where 4-wk old seedlings were grown in pine bark with fertilizer for 4-6 wk. Soybean and pepper seedlings were prepared by growing 9 seeds per pot in sterilized Soilless Potting Mix (Schultz Professional) supplied with fertilizer and fungicide for 2 and 4 weeks, respectively, in the greenhouse. For lupine plants, 10 germinated seeds per styrofoam cup were grown in sterilized vermiculite (Whittemore Com) and fertilizer solution 20-20-20 (Scotts) for 2 wk in the growth chamber.

Single-zoospore inocula with an average concentration of one zoospore per drop (10 μl) were prepared by dilution of a fresh zoospore suspension at 10^4 ^ml^-1 ^with a test solution to 100 zoospore ml^-1^. Test solutions included SDW, dilutions from 1 mM purified AI-2 (Omm Scientific Inc, Dallas, TX) and ZFF from different species. To test whether ZFF was heat or freezing labile, ZFFnic boiled for 5 min or freeze thawed was also included. For determination of the infection threshold of *P. capsici*, the zoospore suspension was diluted in SDW to prepare inocula at 10^2^, 10^3 ^or 10^4 ^ml^-1^, containing an average of 1, 10, or 100 zoospores per 10-μl drop.

For inoculation with *P. nicotianae*, detached annual vinca leaves were used as described previously [18]. Each leaf was inoculated at 10 sites unless stated otherwise with a 10-μl drop of single zoospore inocula. Each treatment included six replicate leaves and was done at least three times.

In the *P. sojae *× lupine phytopathosystem, each cotyledon of lupine plants received one 10-μl drop of a single zoospore inoculum. Each treatment included 10 cups. Each cup contained 5-10 plants. Inoculated plants were kept in a moist chamber at 23°C in the dark overnight, then at a 10 h/14 h day/night cycle until symptoms appeared. Plants with damping-off symptoms were recorded as dead plants. Each assay was repeated twice.

Similarly, for soybean and pepper plant inoculation, two 10-μl drops of an inoculum containing single or multiple zoospores were placed on the hypocotyls of each plant which was laid on its side in a moist chamber. Inoculated plants were kept in the dark overnight and then placed upright in a growth chamber at 26°C until symptoms appeared. For soybean, each treatment included at least 3 replicate pots containing 7-9 plants and was repeated twice. For pepper plants, each inoculation was performed in 6 replicate pots containing 3-8 plants.

### Microscopy of zoospore activity

To determine zoospore responses to ZFF and other chemicals, 30 μl zoospore suspensions at 10^4 ^zoospores ml^-1 ^were added to 120 μl of a test solution in a well on a depression slide to obtain a density of 2 × 10^3 ^zoospores ml^-1^. Test solutions included fresh or treated (boiled or freeze/thawed) ZFF, a serial dilution from purified AI-2 at 1 mM, or SDW. Each test contained two replicate wells per treatment and was repeated once. The slides were placed on wet filter paper in 10-cm Petri dishes and incubated at 23°C. Zoospore behaviors including encystment, aggregation, germination and differentiation in three random fields in each well were examined with an IX71 inverted microscope (Olympus America Inc., Pennsylvania, USA) after overnight incubation. Images were captured with the Image-Pro^® ^Plus software version 5.1 (Media Cybernetics, Inc, Maryland, USA).

### Transient RPI gene silencing mediated by dsRNA and RT-PCR analysis

The procedure was adopted from that for *P. infestans *[41]. To obtain a template for preparation of sense and antisense RNAs by transcription, two pairs of primers containing the T7 RNA polymerase promoter in their forward or reverse sequences were designed for amplification of a partial *RPI *sequence extracted from the *P. capsici *genome http://genome.jgi-psf.org/PhycaF7/PhycaF7.home.html. These primers were dsRPIPcapF: 5'-CAA GCT AAG CAG CTC ATC GCC CA-3'; dsRPIPcapRT7: 5'-GTA ATA CGA CTC ACT ATA GGG CAA CAG GCA CCC CCT GGG TCC A-3'; dsRPIPcapR: 5'-CAA CAG GCA CCC CCT GGG TCC A-3'(TGGACCCAGGGGGTGCCTGTTG); and dsRPIPcapFT7: 5'-GTA ATA CGA CTC ACT ATA GGG CAA GCT AAG CAG CTC ATC GCC CA-3'. Concentrated PCR amplicons were transcribed to produce sense and antisense RNAs using Megascrit RNAi kit (Ambion). Both sense and antisense RNA were mixed to obtain dsRNA at 168 ng μl^-1^.

To silence *RPI*, *P. capsici *protoplasts were transfected with the dsRNA. For each transfection, 24 μl of dsRNA (4 μg) was dried under vacuum (20-30 min) and then suspended in 10 μl PEG and 0.8 M mannitol solutions, respectively then incubated with 10 μl Lipofectin (Invitrogen) for 15 min prior to mixing with 20 μl *P. capsici *protoplasts. Protoplasts were prepared using a modified transformation protocol for *P. sojae *[50]. After further incubation for 24 h at 23°C, the mixture was transferred to 200 ml pea broth with ampicillin and vancomycin then 4 ml was transferred into each well of 12-well plates.

To determine *RPI *expression in dsRNA-treated lines, mycelia from each well (line) were subcultured and extracted for RNA on day 7 using the Qiagen RNeasy plant kit. RNA was prepared from the lines before (T_0_) and two weeks after transfer (T_1_) as well as from the wild type culture. All the RNAs were treated with the RNase-Free DNAase Set (Qiagen), quantified and subjected to reverse transcription using the SuperScript III Reverse Transcriptase kit (Roche) followed by PCR using primers RPIPcapF: 5'- CAG ACG TCG CAG ATA CTA TTA ACC A-3'; and RPIPcapR: 5'-CTC CAG GAA GTA ATG CAT GAC ACA A-3' for *RPI *and actin housekeeping gene primers [50] for an endogenous control. The PCR products were then analyzed by electrophoresis.

### Detection of AHL activity

Acyl-homoserine lactone (AHL) activity was determined with an *Agrobacterium tumefaciens *AHL reporter strain (KYC55/pJZ410/pJZ384/pJZ372) [46]. The reporter strain cannot produce AHLs but has plasmids containing a traI-lacZ reporter fusion and the regulator TraR driven by a T7 expression system. In the presence of exogenous AHLs, the over-expressed TraR activates the reporter fusion, resulting in production of β-galactosidase. The reporter can detect a broad range of AHLs ranging from 4- to 18-carbon acyl moieties at nanomolar levels [46]. We monitored LacZ activity by observing X-gal hydrolysis colorimetrically in the culture plates [51] and quantified the activity using the lactose analog ONPG (orthonitrophenyl-galactopyranoside) in a spectrophotometric assay [46]. ZFFnic and ZFFsoj from different zoospore suspensions, their ethyl acetate extracts, four positive controls (N-hexanoyl-, N-octanoyl-, N-decanoyl-, and dodecanoyl-DL-homoserine lactones (Sigma-Aldrich, Atlanta, Georgia, US) and a negative control (SDW) were included in the experiments. All AHLs were assessed at concentrations of 10 nM and 100 nM.

In plate assays, 10 μl of ZFF, a synthetic AHL or SDW was injected at the center of the test plates with a pipette once the overlay was set. After incubation at 28°C for 2 days, LacZ activity was measured by the diameter of the blue area in test plates. The experiments were performed four times, and each experiment had two replicate plates. In spectrophotometric assays, the reporter was pre-induced in the AT medium containing antibiotics and stored at -80°C. The thawed cells were resuspended in AT medium (1:1000). A 200-μl aliquot of ZFF or SDW, or 50 μl of synthetic AHL was added to glass tubes containing 2 ml suspension. Cultures were grown on a shaker at 28°C until OD_600 _= 1.0 (1.5 days). The bacterial cells in each tube were lysed by the addition of 800 μl of Z buffer, 20 μl of 0.05% SDS and 30 μl of chloroform followed by vortexing. LacZ activity was measured using the Miller Unit at OD_420 _for the supernatant after the reaction with 100 μl of ONPG was ended by 1 M Na_2_CO_3_. The experiment was carried out in replicate and performed twice.

### Statistical analysis

Data from independent experiments were processed and statistically analyzed using ANOVA in Excel. All P-values were determined based on one-way ANOVA unless otherwise stated.

## Authors' contributions

PK conceived of the study, carried out the experiments and drafted the manuscript. BMT identified the RPI gene sequence, participated in designing experiments for *RPI* cloning, silencing and expression, and helped interpret the data and write the paper. PAR maintained cultures of isolates used in all experiments and participated in drafting and editing the manuscript. BWKL conducted chemical analysis of AI-2 in ZFFs and participated in drafting and editing the manuscript. ZSZ has been involved in design and coordination of this study as well as editing of the manuscript. CH participated in conceiving of the study, drafting and editing the manuscript. All authors read and approved the final manuscript.
